# Characterization of a recent malaria outbreak in the autonomous indigenous region of Guna Yala, Panama

**DOI:** 10.1186/s12936-015-0987-6

**Published:** 2015-11-17

**Authors:** José E. Calzada, Ricardo Marquez, Chystrie Rigg, Carlos Victoria, Manuel De La Cruz, Luis F. Chaves, Lorenzo Cáceres

**Affiliations:** Departamento de Parasitología, Instituto Conmemorativo Gorgas de Estudios de la Salud, Panama, Panama; Departamento de Entomología Médica, Instituto Conmemorativo Gorgas de Estudios de la Salud, Panama, Panama; Programa Nacional de Malaria del Ministerio de Salud, Panama, Panama; Institute of Tropical Medicine (NEKKEN), Nagasaki University, 852-8523 Sakamoto 1-12-4, Nagasaki, Japan; Programa de Investigación en Enfermedades Tropicales (PIET), Escuela de Medicina Veterinaria, Universidad Nacional, Heredia, Costa Rica

**Keywords:** Malaria, *Plasmodium*, *Anopheles*, Guna Yala, Panama

## Abstract

**Background:**

This study aims to describe the epidemiological and entomological factors associated with a recent malaria outbreak that occurred in 2012 in a socially marginalized population from Guna Yala Comarca in Panama.

**Methods:**

A descriptive and observational study was conducted by analysing demographic and epidemiological data from all malaria cases registered during 2012 in the Comarca Guna Yala, Panama. Malaria intensity indicators were calculated during the study period. Entomological evaluations were performed monthly, from October to December 2012, in the three communities that presented the most intense malaria transmission during the first semester of 2012. *Anopheles* breeding habitats were also characterized.

**Results:**

During the studied period, 6754 blood smears were examined (17.8 % of the total population), and 143 were confirmed as positive for *Plasmodium vivax.* A significant increase of malaria transmission risk indicators (API: 3.8/1000, SPR: 2.1 %) was observed in Guna Yula, when compared with previous years, and also in comparison with estimates from the whole country. *Anopheles albimanus* was the most abundant and widespread (877; 72.0 %) vector species found in the three localities, followed by *Anopheles punctimacula* (231; 19.0 %) and *Anopheles aquasalis* (110; 9.0 %). Three *An. albimanus* pools were positive for *P. vivax*, showing an overall pooled prevalence estimate of 0.014.

**Conclusions:**

Data analysis confirmed that during 2012 a malaria epidemic occurred in Guna Yala. Panama. This study provides baseline data on the local epidemiology of malaria in this vulnerable region of Panamá. This information will be useful for targeting control strategies by the National Malaria Control Programme.

## Background

As Panamá experiences one of the largest economic growth in Latin America and engages in a national malaria elimination initiative [1, 2], the country still faces a major challenge in controlling malaria transmission among socially marginalized populations [3]. The worst case is that of the ‘Comarcas’: special administrative regions that serve as reservations for Panamanian citizens of Native American descent [4]. These regions and their inhabitants never benefited from sanitation achievements related to the construction of the Panamá Canal and the subsequent development of Panamá [3]. For example, although only 12 % of the total Panamanian population inhabits these reservations, nearly 90 % of the malaria cases in Panamá were reported in these areas during the past decades [1]. Guna Yala, on the Caribbean coast of northeast Panamá is among the poorest regions in Panama, with alarming social and health inequalities compared with other Panamanian provinces [5]. For instance, the multidimensional poverty index (a comprehensive international measurement of acute poverty) was 14.1 % nationwide, but it was 82.3 % in Guna Yala [6]. But more generally, Guna Yala also lags behind in other components of social well-being. For example, it has one of the lowest literacy rates in the country and restricted access to health services [6–8]. Guna Yala’s economy is mainly based on the exchange of subsistence goods, but tourism is now becoming an important part of the economy [6]. More recently, due to its relative geographical isolation the area has been used as an important transit route for illicit South American drugs on their way to US markets [9]. Similarly, the geographical isolation of this region has been a major obstacle to initiate and sustain effective malaria control activities [1]. For example, most communities from this region, where malaria transmission has been reported during the past decade, can be reached only by boats. Other factors, such as languages barriers, a lack of intercultural understanding and political commitment, further complicate any control effort aimed at the Gunas: the main ethnic group in the region, and the one bearing the largest malaria burden in Panamá [3]. All of these factors have resulted in a poor knowledge of the local malaria epidemiology and historical transmission patterns in Guna Yala. In addition, limited evaluation of the currently applied interventions for malaria vector control has been applied in this region.

This study aims to describe the epidemiological and entomological factors associated with a recent malaria outbreak in the Guna Yala Comarca that occurred in 2012. The study also discusses the effectiveness of current control activities executed by the National Malaria Control Programme (NMCP) in this area of Panamá.

## Methods

### Study site and population

**Fig. 1:**
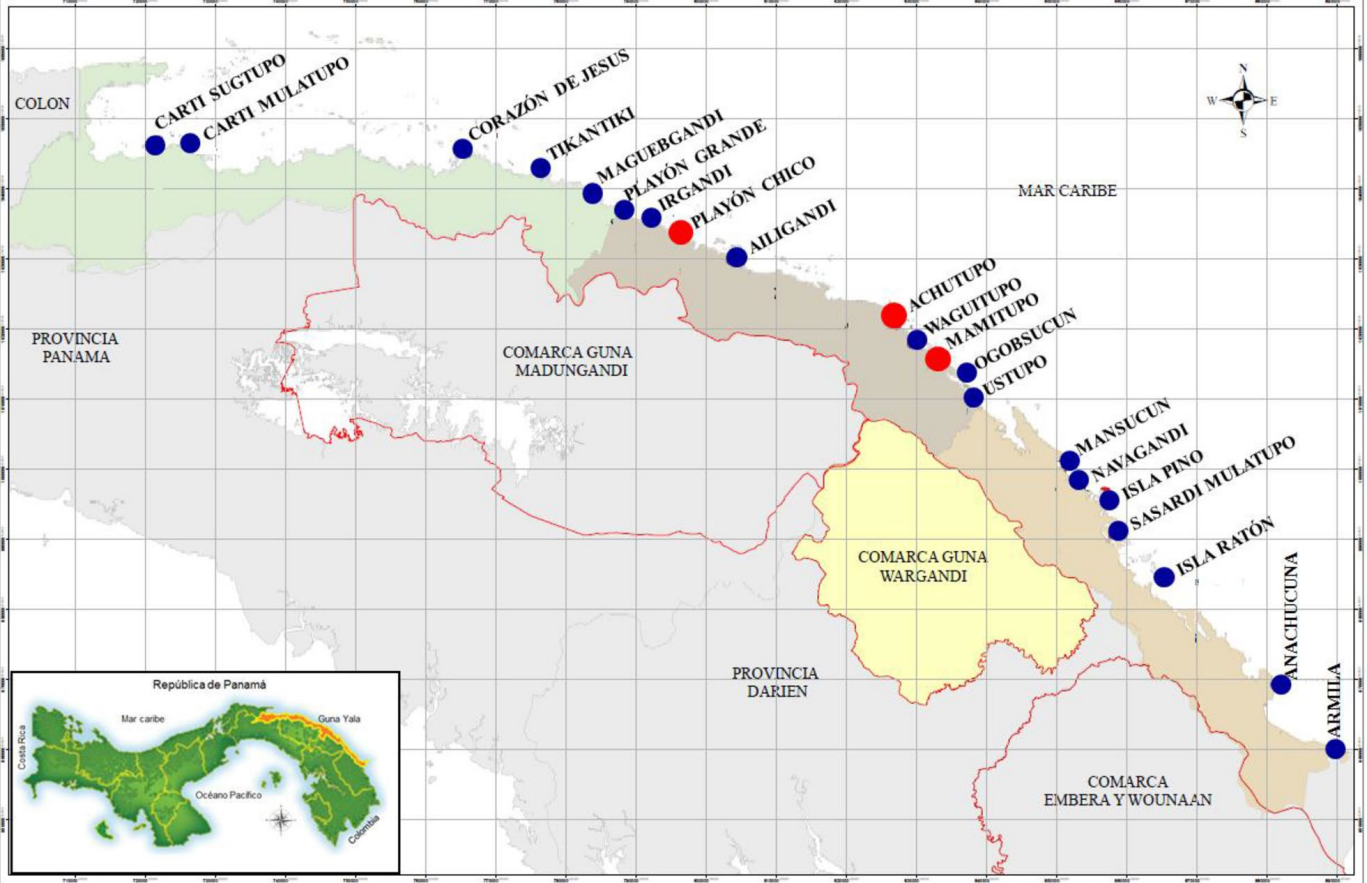
Communities in Guna Yala Comarca in Panama with malaria cases during 2012. Positive communities where entomological surveys were performed are shown in *red circles*

A descriptive and observational study was conducted in the Guna Yula Amerindian autonomous Comarca, located on the Caribbean coast of northeast Panamá, bordering Colombia to the east (Fig. 1). The Guna Yala Comarca comprises around 300,000 ha of continental forest and adjacent coastal waters, including approximately 480 km of coastline surrounded by reefs and mangroves, and around 365 small coral islands, whose ecology has been heavily modified by anthropogenic influence [10]. Previous rainforest, lowland areas on the coastline are now used by Guna people to grow coconuts and other crops, favouring the presence of *Anopheles* spp. mosquito breeding sites.

Guna Yala is politically sub-divided into four *corregimientos* (counties) with 49 *comunidades* (communities) officially recognized, most of which are located on islands near the mainland coast. Each community has its own political organization represented by a political and spiritual leader called ‘*Sahila*’, and the region as a whole is governed by the Guna General Congress [4]. The mean annual temperature, relative humidity and rainfall in Guan Yala are 26.0–27.0 °C, 78.0–90.0 % and 1600–3000 mm, respectively [11]. The region of Guna Yala has normally a unimodal rainfall pattern with a dry season from mid-December to April and a rainy season from May to mid-December.

**Fig. 2:**
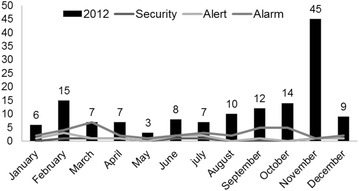
Malaria endemic channel using as reference the monthly *P. vivax* cases registered between 2006 and 2011 in Guna Yala Comarca, Panama

The Gunas have simple lifestyles and maintain their unique traditions. They traditionally sleep in hammocks and their house architecture consists of thatch-roofed huts, with earthen floor and walls made of cane sticks vertically lashed to posts with a fibrous plant. In the larger communities huts are organized into straight streets (Fig. 2). This type of houses do not offer much protection against vectors borne diseases.

### Malaria indicators and data analysis

All malaria cases registered during 2012 in Guna Yala, diagnosed by active or passive surveillance performed by the NMCP from Panamá Ministry of Health (MoH) were analysed. Variables considered in the descriptive analysis were: geographical location of cases, sample collection date, diagnosis date, age, gender, ethnicity, parasite species and density, clinical characteristics of cases, socio-economic status of patients, and time between blood sample collection and diagnosis. Since most of the data was recorded at the population level, only descriptive analysis at the population level, that do not quantify the impact of risk factors, were performed. A quantitative logistic regression was not performed due to the lack of individual based data.

An endemic channel was built using as reference the monthly *Plasmodium vivax* cases registered in Guna Yala in the previous years (2006–2011); 2009 was excluded from this calculation because that year was considered an epidemic year. To estimate the intensity of malaria transmission, the annual blood examination rate (ABER), annual parasite index (API), slide positivity rate (SPR), and the incidence of malaria were also calculated [12]. Demographic and epidemiological data from malaria cases were processed using spreadsheets. Microsoft Excel and Epi Info software were used for analysis. Proportions, central tendency and dispersion measures were calculated.

### Entomological evaluation

Entomological evaluations were jointly performed by the NCMP and Instituto Conmemorativo Gorgas de Estudios de la Salud (ICGES)-trained personnel in the three communities that presented the most intense malaria transmission during the first semester of 2012: Playón Chico (9°18′07.65″N 78°13′29.90″W), Mamitupo (9°11′24.24″N 77°58′24.61″W) and Achutupo (9°11′52.93″N 77°59′13.49″W) (Fig. 1). Collections of immature and adult mosquitoes were performed monthly, from October to December 2012. *Anopheles* spp. mosquito larvae were collected every month during three consecutive days by the dipping method following WHO guidelines [13]. Collections were performed in suspected natural breeding sites near the communities and households in a radius between 1000 and 1500 m (Fig. 2). During larval sampling, the environmental characteristics of each habitat were measured and recorded. Direct landing catches (DLC) of *Anopheles* spp. mosquitoes on humans were performed in the peridomicile from 18:00 to 24:00 h in each locality, once a month for three consecutive days. To minimize the infection risk, catches were performed by trained collectors, following WHO biosecurity guidelines [13]. Outdoor collections were performed simultaneously using a CDC mini-light trap placed near the breeding sites from 18:00 to 06:00 h at 1.5 m. After collection, specimens were placed in labelled cups and transported to the Medical Entomology department at ICGES. *Anopheles* mosquitoes were morphologically identified using taxonomical keys and the reference collection at ICGES.

For both adult and immature mosquitoes, respectively, the human biting rate per night (HBR) and larval density (larvae/sq m) for each species involved in malaria transmission were estimated. To determine the natural infection rates with *Plasmodium* spp. in *Anopheles* spp., adult females were grouped by species and locality. According to the date when they were captured, pools of five specimens were processed to extract DNA using a commercial kit (Blood and Tissue^®^ QIAGEN, Hilden, Germany). The presence of *Plasmodium* DNA was determined with a multiplex-nested PCR as described [14].

### Ethical statement

This outbreak investigation was undertaken by the MoH and ICGES as a joint national surveillance effort to address an immediate and serious threat to public health in Panama. Epidemiological information was obtained from the database available in the NMCP.

## Results

The Guna Yala Comarca has around 37,825 inhabitants distributed in 49 communities, from which 21 (42.8 %) registered malaria cases during 2012 (Fig. 1). Only five of these positive communities were in the mainland. The remainder of the malaria cases were reported in small islands located less than 1 km from the mainland. During the studied period, 6754 blood smears were examined (17.8 % of the total population), and 143 were confirmed as positive for *P. vivax.* Malaria cases were observed in three of the four counties that conform this region: 95 (66.4 %) in Ailigandi, 28 (19.6 %) in Tubualá and 20 (14.0 %) in Nargana. The following communities presented the larger number of cases: Playón Chico (22 cases, 15.4 %), Playón Grande (21 cases, 14.6 %), Mamitupo (21 cases, 14.6 %), Irgandi (ten cases, 7.0 %), Maguebgandi (ten cases, 7.0 %), and Navagandi (nine cases, 6.3 %). Two imported cases (1.4 %) from Colombia were detected, one from the community of Zapzurro and one from Turbo.

An endemic channel was built using as reference the monthly *P. vivax* cases registered in Guna Yala in the previous years, confirming that during 2012 a malaria epidemic occurred in Guna Yala (Fig. 2). During 2012, the months of February (15 cases, 10.5 %), September (12 cases, 8.45 %), October (14 cases, 9.8.0 %), and November (45 cases, 31.5 %) registered the highest malaria incidence (Fig. 2). Indicators (ABER, API and SPR) to monitor malaria levels in Guna Yala (2007-2012) are shown in Table 1. During 2012 a significant increase of malaria transmission risk indicators (API: 3.8/1000, SPR: 2.1 %) was observed in Guna Yula, when compared with previous years, and also in comparison with estimates for the whole country.

**Table 1:** Malaria surveillance indicators in Guna Yala Comarca, Panama, 2007–2012

Malaria indicators	2007	2008	2009	2010	2011	2012	National (2012)
Cases	19	21	115	37	34	143	844
API	0.5	0.6	3.1	1.0	0.9	3.8	0.2
SPR	0.2	0.2	0.9	0.4	0.4	2.1	0.8
ABER	26.5	27.6	33.1	22.5	22.3	17.9	3.1

*API* annual parasite index, *SPR* slide positivity rate, *ABER* annual blood examination rate

During the study period, the median age of malaria cases was 25 years (range 1–74). The under 10 years age group registered the highest number of cases (27.3 %), followed by the 11–20 group (22.4 %) and the 21–30 group (15.4 %) (Table 2). Children under 15 years presented a very high proportional incidence (41.2 %). No cases were recorded in children under 1 year of age. The proportion of male and female cases was similar (51.7 vs 48.3 %) (Table 2).

**Table 2:** Distribution of *Plasmodium vivax* cases by age group and sex registered in Guna Yala Comarca during 2012

Age group In years	Female	Male	Total
0–10	17	22	39
11–20	13	19	32
21–30	11	11	22
31–40	13	8	21
41–50	5	5	10
51–60	6	1	7
>60	4	8	12
Total	69 (48.3 %)	74 (51.7 %)	143

The parasite density observed in the *P. vivax* cases is shown in Table 3. It is noteworthy that 23 cases (16.1 %) presented a parasitaemia higher than 6000/µl. These parasitaemia levels have been associated with severe malaria and poor prognosis for *P. vivax* infections [15–17]. However, no severe malaria cases were reported during 2012 in this region. Of the 143 positive patients, 28 (19.6 %) had detectable *P. vivax* gametocytes. Patients with gametocytaemia were mostly under 40 years old (80 %). All cases presented one or more classic signs for *P. vivax* malaria (Table 4). Patients were successfully treated following NMCP guidelines for malaria treatment, and only presented malaria once during 2012.

**Table 3:** Parasite density range (parasites/µl) observed in *Plasmodium vivax* cases from Comarca Guna Yala in Panama during 2012

Parasitaemia range (parasites/µl)	Number of cases (%)	Accumulated frequency (%)
1–100	15 (10.5)	15 (10.5)
100–500	21 (14.7)	36 (25.2)
500–1000	16 (11.2)	52 (36.4)
1000–2000	16 (11.2)	68 (47.6)
2000–4000	38 (26.6)	106 (74.1)
4000–6000	14 (9.8)	120 (83.9)
>6000	23 (16.1)	143 (100)

**Table 4:** Signs and symptoms frequencies observed in confirmed *Plasmodium vivax* cases from Guna Yala Comarca in Panama during 2012

Signs and symptoms	Frequency (%)
Fever	140 (97.9)
Chills	139 (97.2)
Sweats	128 (89.5)
Headache	68 (47.6)
Arthralgia	135 (94.4)
Myalgia	136 (95.1)
Malaise	131 (91.6)
Vomiting	25 (17.5)
Diarrhoea	49 (34.3)

**Fig. 3:**
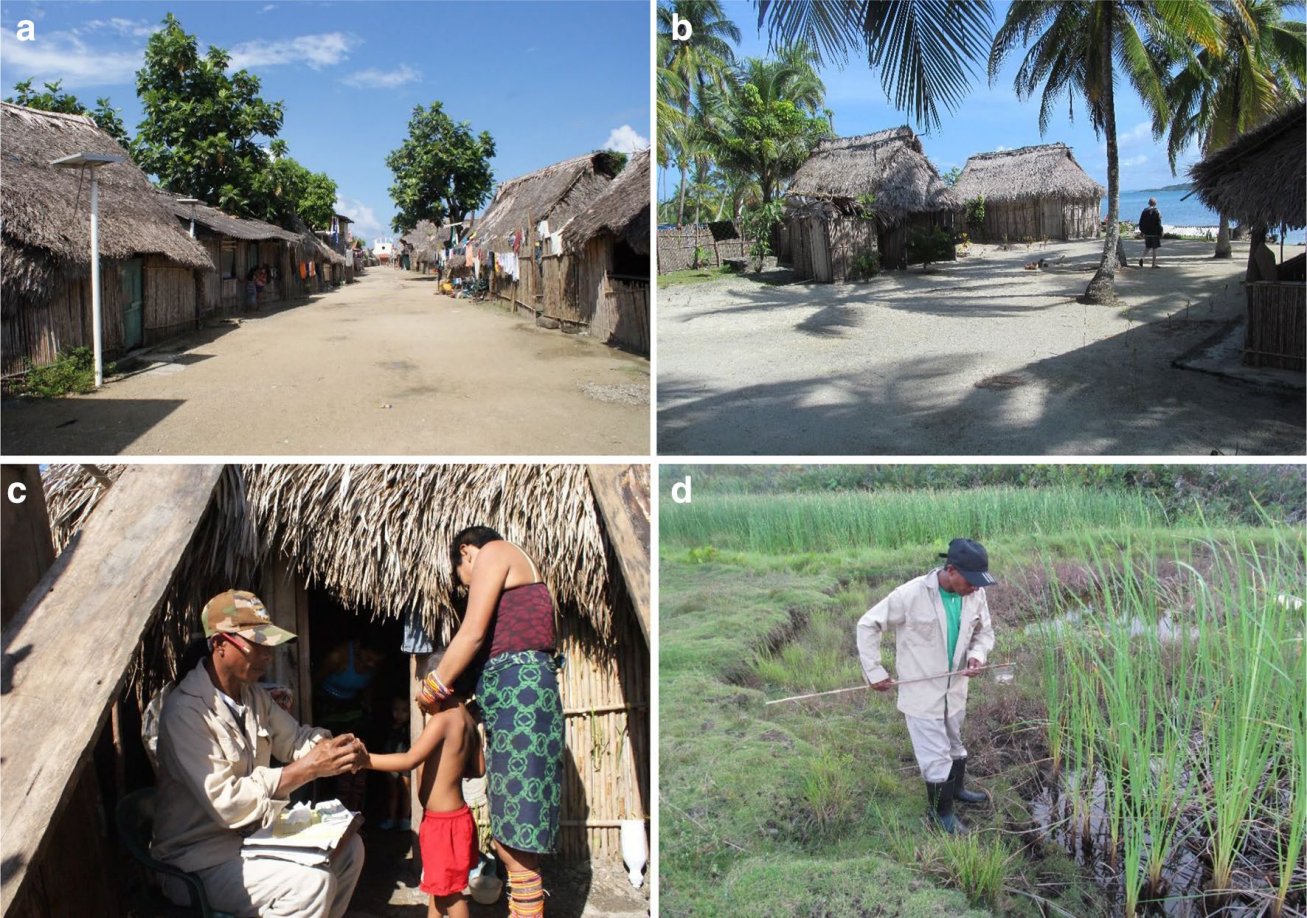
A malaria endemic community in Guna Yala (Playon Chico) showing traditional thatched homes with earthen floor and cane walls. These houses present typical eaves that facilitate *Anopheles* mosquito infestation (**a**, **b**). Active malaria case detection performed by NMCP personnel in endemic areas (**c**). Collection of *Anopheles* larvae from breeding sites near the community of Playon Chico, Guna Yala (**d**)

During the vector survey three predominant types of natural breeding habitats were found, all located in the mainland between 1000 and 1500 m from the three studied communities. In general, habitats were mainly puddles, coastal lagoons or streams, surrounded by emergent vegetation with full or partial sunlight (Fig. 3). A total of 1120 larvae from three different *Anopheles* species were collected and identified in the three localities (Table 5). *Anopheles albimanus* was the predominant vector species with 874 (78.0 %) specimens, followed by *Anopheles punctimacula* with158 (14.1 %) and *Anopheles aquasalis* with 88 (7.9 %). The mean density of larvae collected in the three localities was 8 larvae/sq m (Mamitupo: 11 larvae/sq m; Playón Chico, 7 larvae/sq m; and, Achutupo: 6 larvae/sq m). Detailed estimates by species and location are presented in Table 5. DLC on humans led to the capture of 1218 adult female *Anophelesspp*. belonging to three species (Table 6). *An. albimanus* was the most abundant and widespread (877, 72.0 %), followed by *An. punctimacula* (231, 19.0 %) and *An. aquasalis* (110, 9.0 %). The distribution of collected *Anopheles* spp. mosquitoes by locality and method of collection is shown in Table 6. *An. albimanus* was the species that exhibited the highest HBR in the three localities. Mamitupo was the locality with the highest HBR by *An. albimanus* (15.2 HBR per night) and by *An*. *punctimacula* (3.6 HBR per night), displaying a moderate biting rate for these species. Significant differences between HBR for *An. albimanus* and *An. punctimacula* were observed in the studied localities (Table 6). In general, HBR showed a peak between 18:30 and 19:30 h. CDC traps in the three localities collected significantly fewer mosquitoes than DLC (Table 6).

**Table 5:** *Anopheles* mosquito larvae collected by species and location in Guna Yala Comarca, Panama, 2012

Collected larvae	Locality		
	Playón Chico	Mamitupo	Achutupo
*An. albimanus*			
Number of larvae	159	480	235
Percentage	18.1	55.0	26.9
Average (larvae/m^2^)	14.0	17.0	13.0
*An. punctimacula*			
Number of larvae	42	65	51
% de larvas por	26.6	41.1	32.3
Average (larvae/m^2^)	3.0	3.0	2.0
*An. aquasalis*			
Number of larvae	41	27	20
Percentage	46.6	30.7	22.7
Average (larvae/m^2^)	1.5	1.5	1.0

**Table 6:** Distribution of collected *Anopheles* spp. mosquitoes by locality and method of collection in Comarca Guna Yala, Panama, 2012

Adult *Anopheles*	Locality		
	Playón Chico	Mamitupo	Achutupo
*An. albimanus*	270	365	242
Relative abundance	30.8	41.6	27.6
HBR per night	11.3	15.2	10.1
CDC traps	9	11	8
*An. punctimacula*	75	87	69
Relative abundance	32.4	37.7	29.9
HBR per night	11.3	15.2	10.1
CDC traps	8	7	9
*An. aquasalis*	39	34	37
Abundancia relativa	35.5	30.9	33.6
HBR per night	11.3	15.2	10.1
CDC traps	5	5	7

*HBR* human biting rate

**Fig. 4:**
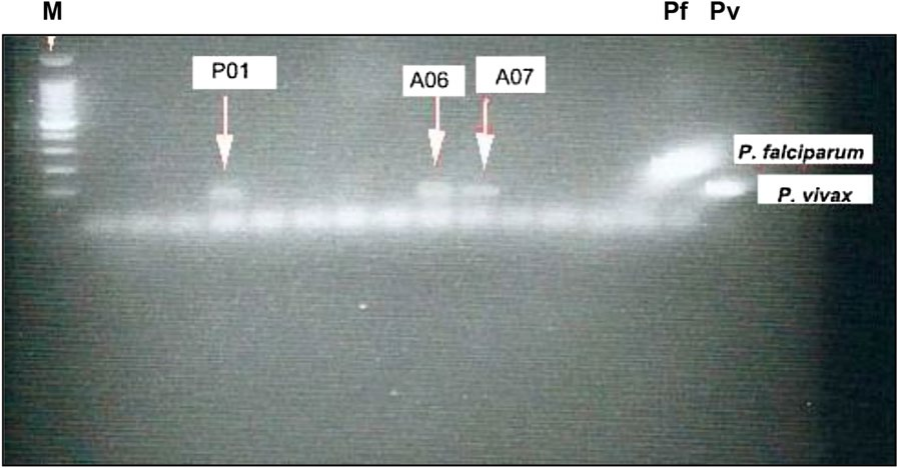
Nested PCR to detect *Plasmodium* infection in *Anopheles* pool samples. M, Molecular size marker (100 bp ladder); P01, A06 and A07, *P. vivax* positive pool samples; Pf, *P. falciparum* positive control; Pv, *P. vivax* positive control

To detect *Plasmodium* infection, 793 adult females were analysed. Forty-four pools from *An. albimanus*, 11 pools from *An. punctimacula* and six from *An. aquasalis* were processed for PCR analysis. Three *An. albimanus* pools (two from the community of Achutupo and one from Playón Chico) were positive for *P. vivax* (Fig. 4), showing an overall pooled prevalence estimate of 0.014. Samples analysed from *An. punctimacula* and *An. aquasalis* were negative for *Plasmodiumspp.*

## Discussion

Panamá is one of the eight countries that conformed the Mesoamerica region (Southeast Mexico States and all Central American countries). The significant reduction observed recently in most countries of this region has motivated the launch of an initiative for Malaria Elimination in Mesoamerica and Hispaniola with the active involvement of the National Malaria Control Programmes of nine countries and the support of the Global Fund for Aids, Tuberculosis and Malaria [18]. National political support for this goal was also evidenced by a recent resolution passed by the ministers of health of the Mesoamerican countries as well as the Dominican Republic and Haiti to eliminate malaria by 2020 [19]. Four Mesoamerican countries are currently in the pre-elimination phase (Mexico, Belize, Costa Rica, and El Salvador). Only Nicaragua and Panamá reported an increase in malaria cases from 2011 to 2012 [20].

The National Malaria Control Programme of Panamá guides and coordinates all malaria control activities in the country [21]. Prevention and control activities are mainly focused on early diagnosis that relies on microscopy and on prompt treatment. Malaria cases are mostly detected by active surveillance performed by NMCP personnel in endemic areas. Health facilities conduct passive case detection without malaria-specific screening centers. These services are free of charge. Chloroquine in combination with primaquine is used for *P. vivax* malaria treatment. Panama is the only country in Central America that recommends artemisinin combined therapy for the treatment of *P. falciparum* infections. Although autochthonous *P. falciparum* infections have not been reported in Panama during the past years, there is an increasing detection of imported cases. Vector control activities routinely performed by the NCMP in Panamá are basically limited to indoor residual spraying (IRS) performed in areas that are considered at risk for malaria transmission based on the malaria incidence observed in previous years. However, due to their traditional beliefs and practices, IRS is still not well accepted by Gunas, with coverage reaching frequently less than 50 % of the households in any community [22]. Interestingly, malaria cases were recorded in nine of the 14 communities where IRS was performed during 2012, underlining the need to either increase IRS coverage and to identify different control strategies that are accepted by Guna communities. It is also important to evaluate the possible resistance of the local vectors to the insecticides currently used by the NMCP, an activity that is not systematically performed in the country for malaria or any other vector-borne diseases. Fogging with deltamethrin is occasionally used as a vector control intervention during epidemics [21].

Malaria control in border areas has been a challenge for the NMCP in Panamá. In fact, malaria transmission is currently focused in two regions near the borders that together occupy 38.6 % of the country’s territory. The eastern focus, close to the border with Colombia, is represented by the indigenous Comarcas of Guna Yala, Madungandí and Wargandi, and the province of Darien. The western focus, close to the border with Costa Rica, is represented by the province of Bocas del Toro and the Comarca of Ngäbe Bugle.

From 1965 to 2012, 8.9 % of the total malaria cases in Panamá were reported in Guna Yala. In this region malaria transmission is considered epidemic-prone, associated with the movement of infected Gunas across the region, as well as the frequent presence of temporal immigrants from endemic-malaria areas in South America [3, 23]. Once malaria is established in Guna Yala, the culturally driven migrations of the Gunas favour the spread of the disease to other indigenous regions of the country occupied by this ethnic group, such as Wargandi and Madungandi in the eastern half of Panamá [3].

Most communities in Guna Yala are highly vulnerable to malaria transmission, mainly because of the continuous presence of suitable breeding habitats for *Anopheles* near dwellings, which are also mosquito friendly, having no eaves that stop *Anopheles* spp. infestation [24, 25]. Other risk factors such as destitute housing, which is inappropriate for residual insecticide application [26], and the rejection by the Gunas of different vector control measures, also contribute to the vulnerability of this region [22]. In fact, recent studies have shown that traditional house architecture found in Gunas communities is a risk factor for malaria transmission [23, 27]. In this sense, any effort for housing improvement requires multidisciplinary research, and particularly a multicultural approach that considers the traditional beliefs and practices of Guna communities [22].

During 2012, malaria transmission in this region was continuous. However, the largest number of cases was observed during the last three months of 2012 (Fig. 1), probably associated with rainfall and agricultural, commercial and migration activities that normally increase at the end of the year [28]. In this sense, malaria is perceived as one of the vector-borne diseases more likely to be affected by global climatic changes [29, 30]. For instance, temperature and humidity are factors that affect mosquito longevity and the rate of oogenesis, consequently increasing the potential for malaria transmission [31–33].

It was recently described that changes in climatic variability were significantly related to malaria transmission in the Madungandi Reservation, a region also inhabited by Gunas in Eastern Panama [3]. Due to its location and geographic characteristics (an extended coastline and many inhabited small islands), the Guna Yala region is highly vulnerable to weather-related events. There is strong evidence of an important increase in sea level and a reduction in surface area from uninhabited islands in Guna Yala [10], causing the displacement of some Guna populations from the islands to mainland areas, where there is a higher risk for malaria transmission [4]. This situation highlights the need to study the contribution of climate change in the dynamics of malaria transmission in this vulnerable region from Panamá.

During the past decades the intensity of transmission and the risk of malaria infection have shown significant spatial and temporal fluctuations in Panamá. In this regard, it is important to estimate accurately the malaria burden in each region for an appropriate planning of control interventions, as well as for a more rational allocation of existing resources to control malaria. The API, SPR and ABER are malaria surveillance indicators usually estimated for this purpose [34, 35]. At the end of 2012, the API in Guna Yala was 3.8/1000 inhabitants, which represents a 4.2 times increase compared with the one observed the previous year (0.9/1000 inhabitants). In many countries the SPR has been used as a predictor of malaria incidence and as an important indicator to evaluate malaria control programmes [34, 35]. In 2012, the SPR in Guna Yala was very high (2.1 %) compared with the national statistics of 2012 (0.3 %), corresponding to the increase in malaria incidence observed in this region during 2012. The ABER was 17.4 %, indicating the examination of a large number of blood smears in search of suspected malaria cases (Table 1). This last indicator estimates the operational efficacy of the NMCP.

When *P. vivax* cases were stratified by gender, a slight predominance, although not significant, of males over females was found (51.7 vs 48.3 %). In this region, male Gunas travel from their work to their homes during the first hours of the evening and they also frequently engage in night recreational activities, factors that favour the exposure to infective malaria bites by mosquitoes. It is noteworthy that 90 % of cases were ≤40 years old, and about half (47.6 %) of the cases were in the economically active population, between 15 and 50 years old (Table 2).

Early diagnosis and prompt treatment are crucial factors to consider for malaria control success. Indeed, starting treatment before the appearance of gametocytes is a key strategic point in the interruption of transmission [36]. During the study period, the median time between blood sample collection and thick-smear diagnosis was very high (7 days, range 1–20 days), a factor that most likely was decisive for the high transmission and dispersion of malaria in this region during 2012. In this regard, it is key to highlight the importance of implementing rapid diagnostic tests (RDT) as part of the routine activities of the NMCP, especially during outbreaks occurring in areas of difficult access with scarce health infrastructure, as is the region of Guna Yala. During the past years the MoH of Panama, with the support from PAHO, has undertaken significant efforts to train field personnel and to validate different RDTs available in the market. However, due to legal issues with the Panamanian Medical Technologists Association it has so far not been possible to implement this diagnostic methodology for routine malaria diagnosis by the vector inspectors from the NMCP.

No severe *P. vivax* malaria cases were reported during this period, despite the high parasitaemias observed in many cases. All patients responded adequately to the national standard treatment for vivax malaria [21], and no relapses were recorded during the study period. However, the NMCP believes that within the Guna indigenous population and/or from frequent temporal visitors from South America, there are asymptomatic reservoirs contributing to maintain malaria transmission in the area, a fact that is being reported with growing frequency in Latin America [37]. In this sense, a more comprehensive sampling and the use of more sensitive molecular methods would have been needed to identify asymptomatic infections and determine the real malaria burden in the study area during the epidemic.

In the line with previous studies conducted in Panama [38, 39], *An. albimanus* was by far the predominant species in the study area, exhibiting the highest prevalence and HBR in the three localities. It was also the only species found naturally infected with *P. vivax*, confirming the importance of *An. albimanus* as a major malaria vector in this region of Panamá, and most likely responsible for human malaria transmission during the 2012 epidemic. *An. albimanus* is a species that breeds in a wide variety of aquatic habitats with several types of vegetation in their habitats [40, 41], and is considered a major malaria vector throughout Latin America [42, 43].

*Anopheles punctimacula* and *An. aquasalis* were found in much smaller numbers. Both species have been incriminated as secondary vectors of human malaria in Panama [44], and have been previously described at different densities in Guna Yala [38]. *An. punctimacula* showed preference for shallow waters shaded by coconut palms, breeding characteristics described in earlier studies for this species [39, 42]. *Anopheles aquasalis*’ breeding sites were mangroves and coastal wetlands. Its abundance has been associated with salinity, the presence of aquatic vegetation and permanent breeding sites [45–47]. *An. aquasalis* has a restricted distribution in Panamá, being particularly prevalent in Guna Yala [38]. In fact, in some communities of this region it has been reported at higher rates than *An. albimanus*. *Anopheles aquasalis* is also considered an important vector of malaria in many countries from Latin America [43]. For these reasons it has been suggested that *An. aquasalis* may play an important role for local malaria transmission in the study region [38]. However, although *An. aquasalis* was found in entomological surveys at the three collection sites, it had a low density. Moreover, *Plasmodium* infection was not detected in this mosquito species, although more detailed entomological studies are necessary to better understand any vectorial role for this species.

It is important to note that many of the inhabited islands in Guna Yala lack fresh water, thus Gunas have settled on those islands that are closest to the mainland. The mainland is where they farm crops, hunt and access water from rivers, while on the islands they live and fish on the ocean [48]. Interestingly, during the study period no breeding sites or infected *An. albimanus* mosquitoes were found in the inhabited islands. Both were found on mainland about 1.0 km from the communities.

An important limitation of the entomological evaluation is that *Anopheles* collections were performed only during the last 3 months of 2012 and when a malaria epidemic was occurring in the region. The entomological findings may, therefore, be biased, complicating their interpretation and comparison with other studies.

During the study period, two imported malaria cases were detected from nearby endemic communities on the Atlantic coast from Colombia, where both *P. vivax* and *Plasmodium falciparum* circulate. The frequent movement of Gunas and non-indigenous populations between the Panamá-Colombia border is a constant threat, and was most likely the cause of the introduction in 2002 of chloroquine-resistant *P. falciparum* parasites in Guna Yala, that spread to other Guna regions in eastern Panamá [49, 50]. This possibility has been recently reinforced by the molecular relatedness between Colombian and Panamanian malaria samples. Molecular barcoding and drug-resistant loci profiles suggest a northward movement of drug-resistant *P. falciparum* parasites along the Atlantic coast [51]. The region of Turbo in Colombia, from where one of the imported cases came, deserves special attention as not only *P. falciparum*-resistant parasites circulate in this area, but also clinical resistance of *P. vivax* to chloroquine has been reported [52]. This situation calls for an urgent and efficient cross-border cooperation where both neighbouring countries engage in the elimination process, following the example of the concerted efforts between Costa Rica and Panamá in the western border when dealing with malaria control in the migrating Ngabe-Bugle [3, 39].

## Conclusion

This study provides baseline data on the local epidemiology of malaria in this vulnerable region of Panamá. This information will be useful for targeting control strategies by the NMCP. This study also described some of the complex issues that need to be solved in order to achieve malaria elimination in the context of a highly mobile and marginalized population with strong cultural traditions and beliefs.

## Abbreviations

ABER: annual blood examination rate
API: annual parasite index
DLC: direct landing catches
HBR: human biting rate
ICGES: Instituto Conmemorativo Gorgas de Estudios de la Salud
IRS: indoor residual spraying
MoH: Ministry of Health
NMCP: National Malaria Control Programme
PAHO: Pan American Health Organization
RDT: rapid diagnostic test
SPR: slide positivity rate
WHO: World Health Organization
